# Comparison of Compressive Stress-Relaxation Behavior in Osteoarthritic (ICRS Graded) Human Articular Cartilage

**DOI:** 10.3390/ijms19020413

**Published:** 2018-01-31

**Authors:** Rajesh Kumar, David M. Pierce, Vidar Isaksen, Catharina de Lange Davies, Jon O. Drogset, Magnus B. Lilledahl

**Affiliations:** 1 Department of Physics, Norwegian University of Science and Technology (NTNU), N-7491 Trondheim, Norway; catharina.davies@ntnu.no (C.d.L.D.); magnus.lilledahl@ntnu.no (M.B.L.); 2 Departments of Mechanical Engineering/Biomedical Engineering/Mathematics, University of Connecticut, Storrs, CT 06269, USA; dmpierce@engr.uconn.edu; 3 Department of Clinical Pathology, University Hospital of Northern Norway, N-9038 Tromsø, Norway; vidar.isaksen@unn.no; 4 Department of Orthopaedic Surgery, Trondheim University Hospital and Norwegian University of Science and Technology (NTNU), N-7491 Trondheim, Norway; jon.o.drogset@ntnu.no

**Keywords:** stress-relaxation, polymer dynamics, biomechanical characterization, articular cartilage, osteoarthritis

## Abstract

Osteoarthritis (OA) is a common joint disorder found mostly in elderly people. The role of mechanical behavior in the progression of OA is complex and remains unclear. The stress-relaxation behavior of human articular cartilage in clinically defined osteoarthritic stages may have importance in diagnosis and prognosis of OA. In this study we investigated differences in the biomechanical responses among human cartilage of ICRS grades I, II and III using polymer dynamics theory. We collected 24 explants of human articular cartilage (eight each of ICRS grade I, II and III) and acquired stress-relaxation data applying a continuous load on the articular surface of each cartilage explant for 1180 s. We observed a significant decrease in Young’s modulus, stress-relaxation time, and stretching exponent in advanced stages of OA (ICRS grade III). The stretch exponential model speculated that significant loss in hyaluronic acid polymer might be the reason for the loss of proteoglycan in advanced OA. This work encourages further biomechanical modelling of osteoarthritic cartilage utilizing these data as input parameters to enhance the fidelity of computational models aimed at revealing how mechanical behaviors play a role in pathogenesis of OA.

## 1. Introduction

Articular cartilage is the tissue that covers the bone ends in diarthroidal, synovial joints. It acts like a cushion between the ends of two bones, minimizes the stress by distributing loads, and with very low frictional properties facilitates smooth joint movement. The mechanical properties of articular cartilage depend on the structural integrity of the constituents of the matrix composed mainly of collagen, proteoglycan (PG) and water [1].

Osteoarthritis (OA) is the most prevalent musculoskeletal disorders among elderly people [2] and is one of the fastest increasing causes of individual and social economic burden [3]. It is articular cartilage that is primarily affected during the progression of OA. The event and stimuli that initiate cartilage degradation is still unknown. Early degenerative changes in articular cartilage associated with OA are most often not visible by standard clinical imaging system (e.g., X-ray, MRI, Ultrasound, Arthroscopy). Our previous studies have shown biochemical and structural changes in early stage of osteoarthritic cartilage [4,5,6]. In addition to the biochemical and structural changes, the biomechanical properties of the articular cartilage have been shown to evolve with the stage of OA [7,8,9,10,11]. Current understanding about the changes in biomechanical properties and its consequences in cartilage degradation during progression of OA is limited. Therefore, knowledge about the changes in mechanical behavior of articular cartilage at various stages of OA can help in understanding the mechanisms of disease progression.

Previous studies relevant to the biomechanical characterization of osteoarthritic articular cartilage were performed using normal and/or degenerative cartilage [10,11,12,13,14,15,16,17]. The International Cartilage Repair Society (ICRS) grade is a clinical, standard grading system used by orthopaedic surgeons to evaluate morphologically the degeneration of osteoarthritic cartilage [18]. More precise investigations with use of different stages of human osteoarthritic cartilage, i.e., the inclusion of early (ICRS grade-I), intermediate (ICRS grade-II) and, advanced stage (ICRS grade-III) osteoarthritic cartilage in biomechanical characterization is seldom. However, Klemann et al., showed the creep behavior characteristics of progressively osteoarthritic cartilage [7]. The investigation of stress-relaxation behavior can help further in characterizing the viscoelastic behavior of osteoarthritic cartilage.

In this study intact ex-vivo cartilage sections were characterized by mechanical indentation. The stress-relaxation data acquired were fitted to the stretch exponential model that is described elsewhere [19,20,21,22]. This stretch exponential model is an empirical approach that describe relaxation processes for a wide variety of systems including polymers [16,20,21,22]. The cartilage matrix is composed of several molecules that provide varying molecular environments, resulting in an overlap of many relaxation rates often observed as stretched exponential kinetics. The model represents the polymeric mechanism of cartilage viscoelasticity. This is a time-dependent viscoelastic model suitable for the unconfined compression and long-term behavior analysis, the environment under which the data were acquired. In the stretch exponential model, stress relaxation is described by

(1) $$\sigma =\left(\sigma_{\mathrm{in}}-\sigma_{\mathrm{eq}}\right)e^{-\left(t/\tau \right)\beta }+\sigma_{\mathrm{eq}}$$

where, $\sigma_{\mathrm{in}},\;\sigma_{\mathrm{eq}}$, *τ*, and *β* is the instantaneous stress, equilibrium stress, time constant, and the stretching exponent, respectively. The stress relaxation time constant (*τ*) and stretching exponent parameter (*β*) were determined by nonlinear curve fitting using Matlab^®^ (The MathWorks, 2014, Natick, MA, USA). The stress-relaxation time constant represents the viscoelastic characteristic of the cartilage while the stretching exponent parameter represents the distribution of relaxation time and is related to the specific type of polymer motion e.g., reptation.

In this work, we hypothesized that the parameters derived from the stretch exponential model can describe the biomechanical characteristics of osteoarthritic articular cartilage. Therefore, the aim was to compare the stress-relaxation constant (*τ*) and the stretching-exponent parameter (*β*) between ICRS grades I, II and, III and find any relation, if existing, with the biochemical content.

## 2. Results

**Figure 1:**
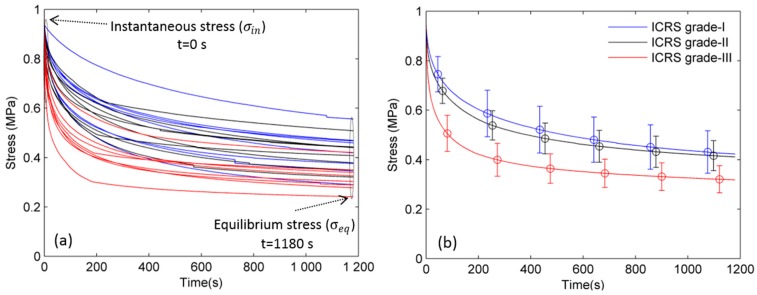
(**a**) Stress-relaxation data acquired from 24 cartilage sections; (**b**) Mean stress-relaxation curve (*n* = 8 curve) of ICRS grades I, II, and III. Bars represent the standard deviation at the selected time points.

**Figure 2:**
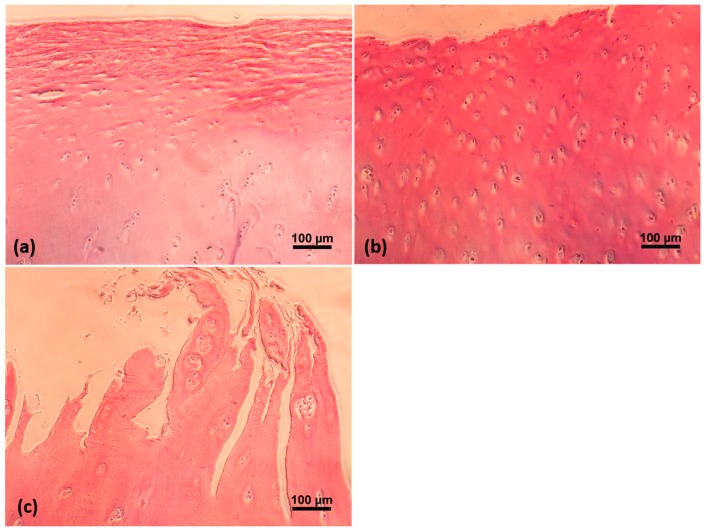
Histological images of osteoarthritic articular cartilage stained by Hematoxylin and Eosin (H&E). (**a**) ICRS grade-I; (**b**) ICRS grade-II; (**c**) ICRS grade-III.

The stress relaxation data acquired from 24 cartilage sections are shown in Figure 1a. A statistically significant and high correlation was observed between the ICRS and OARSI grades (*R*^2^ = 0.682, *p* < 0.05). Therefore, histological evaluation (OARSI grade) confirmed the progressive degradation of osteoarthritic cartilage in addition to the macroscopic evaluation (ICRS grade) performed by the orthopaedic surgeons (Figure 2).

A comparison between the mean relaxation curves of ICRS grades I, II, and III is shown (Figure 1b). The difference in mean relaxation behavior of different ICRS grades of osteoarthritic cartilage was observed. To measure a quantitative difference, the stress-relaxation curves were fit to Equation (1). The results obtained are summarized in Table 1.

### 2.1. Young’s Modulus

**Figure 3:**
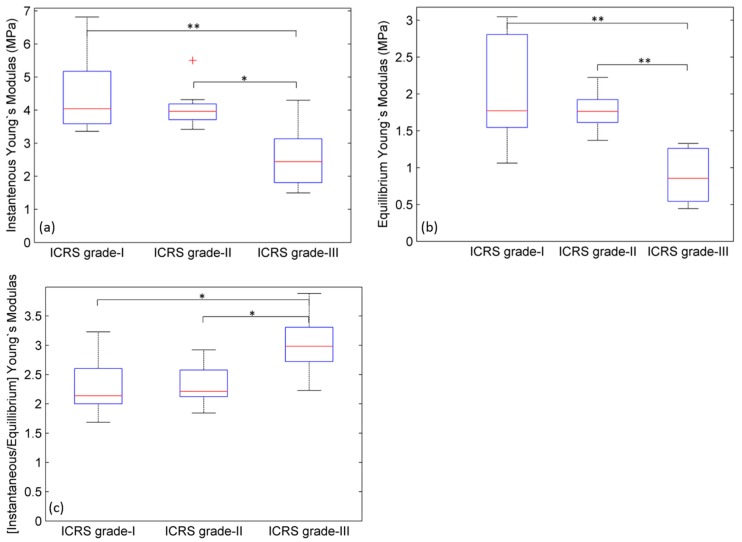
A relative comparison of (**a**) instantaneous Young’s modulus (Y_in_) and (**b**) equilibrium Young’s modulus (Y_eq_) between three ICRS grades of human osteoarthritic cartilage. Significant reduction in ‘Y_in_’ and ‘Y_eq_’ between grade-I and -III and, grade-II and -III were observed. No significant change between grade-I and -II was observed; (**c**) A relative comparison of Y_in_/Y_eq_ between three ICRS grades of human osteoarthritic cartilage. The Young’s modulus ratio represents the vitality of cartilage (the smaller the value, the more healthy the cartilage). Two asterisks ** represent *p* < 0.01 and one asterisk * represents *p* < 0.05. + represent the outliers.

Although the same load was applied to all cartilage sections, the induced deformations (i.e., strains) due to the applied load were observed to be different in different ICRS grades of osteoarthritic cartilage. Analysis of the Young’s modulus shows that there is a significant (*p* < 0.05) decrease in Y_in_ (Figure 3a) and Y_eq_ (Figure 3b) values between grades I and III, and between grades II and III, but no significant (*p* < 0.05) difference observed between grades I and II. The magnitude of Young’s moduli ratio (i.e., Y_in_/Y_eq_) was evaluated and compared between ICRS grades I, II and III (Figure 3c).

### 2.2. Stress Relaxation Time (τ)

**Figure 4:**
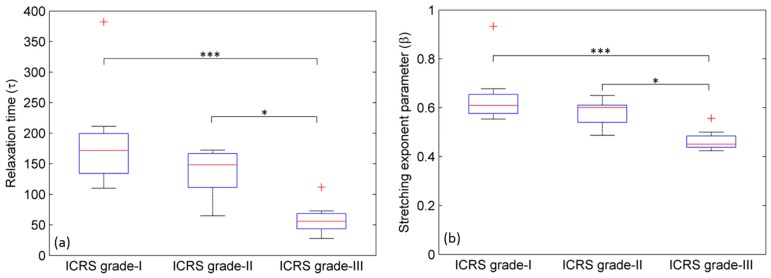
A relative comparison of (**a**) stress relaxation times (*τ*) and (**b**) stretching-exponent parameter (*β*) between three ICRS grades of human osteoarthritic cartilage. Significant reduction in ‘*τ*’ and ‘*β*’ between grade-I and -III and grade-II and -III were observed. No significant reduction between grade-I and -II was observed. Three asterisks *** represent *p* < 0.001 and one asterisk * represents *p* < 0.05. + represent the outliers.

A comparison of stress-relaxation times (*τ*) between three ICRS grades I, II and III was performed. Figure 4a shows that the median value of the stress relaxation time (*τ*) decreases with the ICRS grade. The difference was statistically significant (*p* < 0.05) between grades I and III and between grades II and III but not between grades I and II.

### 2.3. Stretching Exponent Parameter (β)

A relative comparison of the stretching exponent (*β*) between the three ICRS grades revealed a statistically significant (*p* < 0.05) decrease between grades I and III and between grades II and III but not between grades I and II (Figure 4b).

## 3. Discussion

### 3.1. Young’s Modulus

In our previous study, a comparison among ICRS grades of osteoarthritic cartilage was reported that showed a significant reduction in PG content between grades I and III, and between grades II and III, but not between grades I and II [5]. This means that the change in Young’s modulus shows the same trend as the change in PG content between the ICRS grades I, II, and III. Earlier studies have shown that there is a strong positive correlation between compressive stiffness and the PG content of cartilage [23,24]. Therefore, our result agrees with the earlier findings that the compressive modulus in cartilage is related to the PG content in the extracellular matrix (ECM) of the cartilage [23,24].

In advanced stage (ICRS grade-III) of osteoarthritic cartilage, the lowest Yin was observed due to the highest induced deformation. The resistance to the deformation of tissue depends on the interactions between macromolecules and fluid-flow through the cartilage matrix. Using an OA model, Mansour et al. [25], has described that decreasing PG content allow more space in the tissue for fluid and subsequently lower resistance to flow in the advanced stages of OA in cartilage. Therefore, the lowest PG content in ICRS grade-III causes the highest induced deformation. This explains why lowest Y_in_ is observed in the ICRS grade-III.

The Young’s modulus ratio has been used to assess the vitality of cartilage [10,26]. The term vitality indicates the tissue’s mechanical strength and ability to sustain loading and unloading processes under normal functioning of articular cartilage. The smaller the ratio, the more viable the cartilage. The Young’s modulus ratio (Y_in_/Y_eq_) was found to be highest in ICRS grade-III (Table 1).

Using the creep test on articular cartilage, Kleeman et al. reported a reduction in Young’s modulus with progressive ICRS grade [7]. However, neither the stress-relaxation test (in the present analysis) nor the dynamic impact test reported by Kos et al. [27] observed a significant difference in Young’s modulus between ICRS grades I and II.

### 3.2. Stress Relaxation Time (τ)

It was observed that the stress-relaxation time (*τ*) in ICRS grade-III cartilage is ~2 times shorter than the ICRS grade-I (Table 1). However, no significant difference was observed between ICRS grade-I and ICRS grade-II. Therefore, we hypothesize that in the advanced stage (i.e., ICRS grade-III), a significant decrease in PG content allows more space in ECM for fluid. An increase in water content correlates with an increase in permeability. Increasing permeability allows fluid to flow out of the cartilage matrix more easily and rapidly, resulting in a lower-stress relaxation time [28,29]. Moreover, polymer dynamics theory predicts that a decrease in the stress-relaxation time constant and a loss in modulus (stiffness) occurs due to the decrease in average molecular length of the polymer [30]. June et al. showed a decrease in relaxation time by cleaving the collagen triple-helix molecule at multiple locations and cleaving hyaluronan but not glycosaminoglycans [19,31,32]. Taking both effects into the account, we believe that the decrease in average molecular length of the polymer (i.e., degradation in collagen fibers) and the reduction in proteoglycan content are together responsible for a significant decrease in stress-relaxation time in the advanced stages of OA.

### 3.3. Stretching Exponent Parameter (β)

The parameter *β* represents the width of the of relaxation-time distribution. The numerical value of *β* lies between 0 and 1. A narrow distribution has a *β*-value close to 1, while wider distributions result in lower *β* values. Physically, *β* is associated with a specific type of polymer motion called reptation. Fyhrie et al. showed that the stress-relaxation response of cartilage follows the reptation dynamics of the polymers [33]. However, which biopolymer (chondroitin sulfate, proteoglycan, or hyaluronic acid polymer) play dominant roles in the reptation was not described. Following this study, Ruberti et al. determined that the reptation dynamics in cartilage is mainly due to the hyaluronic acid chains and not due to proteoglycan and chondroitin sulfate side chains that are attached to them [34]. These side chains are sufficiently stiff so that their retraction times are very short [30] and hence their contribution to reptation is not significant [34]. This indicates that there is a significant loss of hyaluronic acid accompanied by the loss of PG in advanced stage OA. In the ECM of cartilage, PG molecules are attached to the long backbone chain of the hyaluronic acid. Therefore, significant loss in hyaluronic acid polymer might be a reason for the loss of PG in advanced OA.

The result is a speculation which is based on the earlier published results [30,33,34]. To validate the result, further biochemical assessment is required. Normal (healthy) human cartilage samples were not available during this study. However, collection of such samples during autopsy are on the way and, our future investigation would allow to compare the results with healthy cartilage.

## 4. Materials and Methods

### 4.1. Sample Preparation

The use of human samples was approved (2013/265 REK, 7 March 2013, Norway) by the Regional Committee for Medical Research Ethics. Articular cartilage samples were obtained from osteoarthritic patients undergoing total knee replacement surgery. It was confirmed that no patient had suffered prior knee injury or surgery. Eight cartilage sections of each ICRS grade I, II, and III were obtained. Thus, in total 24 sections of articular cartilage (from 14 patients) were entered into the study. The contribution of each patient in the collection of cartilage sections is shown in Table 2. To reduce age-associated variation in the articular cartilage, all patients selected in this study were older than 65 years. Any two cartilage sections presenting the same ICRS grade from the same patient were not included in this study. ICRS grade-IV usually includes only remnants of cartilage and mostly exposed bone and therefore, was not included in the study. Cartilage sections were acquired from the femoral condyle of the knee during arthroplasty. The assignment of ICRS grade was performed by two experienced orthopedic surgeons, who were blinded to the classification of each other. Only samples assigned the same ICRS grade by both orthopedic surgeons were included in this study. The cartilage tissues were wrapped in phosphate-buffered saline (PBS)-soaked cotton to avoid dehydration and stored at 4 °C. Each stress-relaxation measurement was completed within 24 h of harvesting the tissue. Before the measurements each cartilage tissue was dissected by a surgical scalpel, perpendicular to the articular surface in a cubical shape having side approximately 6 mm and was immersed in PBS for approximately 30 min at room temperature.

### 4.2. Data Acquisition and Analysis

**Figure 5:**
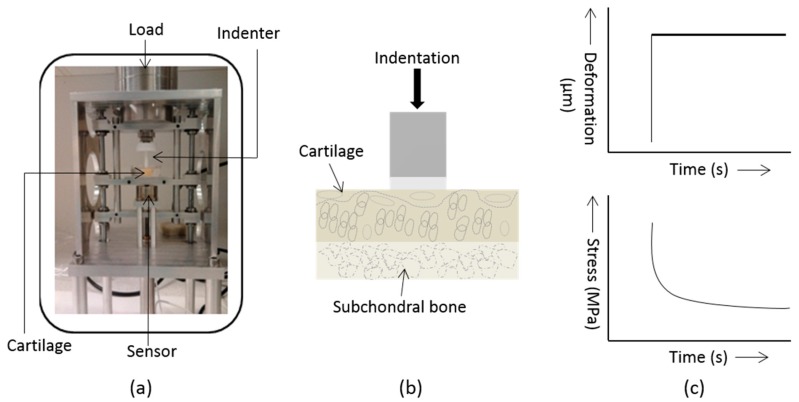
(**a**) A photograph of indentation instrument; (**b**) Indentation was imposed on articular surface of cartilage by a preload; (**c**) Deformation of articular surface due to applied load and acquisition of stress-relaxation data.

The stress-relaxation tests were performed uniaxially by a macroscopic indentation device developed in the laboratory (Figure 5a). A deformation equivalent to a load of 1.2 kg was suddenly imposed on the articular surface of the cartilage section (Figure 5b). Due to the fixed, applied deformation, a transient decrease (i.e., relaxation) in compressive force (and therefore stress) in the cartilage matrix occurs that was captured with a load cell (S/N: 1258426, Honeywell Sensotec Sensors, USA). The deformation on the articular surface of cartilage was imposed by using a flat-ended, cylindrical, impermeable indenter of diameter 4 mm (Figure 5b). The stress-relaxation data were acquired for 1180 s (Figure 5c). The initial stress-relaxation was set to *t* = 0 s and termed instantaneous stress ($\sigma_{\mathrm{in}}$) while the stress-relaxation measured at *t* = 1180 s was set as the equilibrium stress ($\sigma_{\mathrm{eq}}$). The instantaneous Young’s modulus (Y_in_) and the equilibrium Young’s modulus (Y_eq_) are the point measurements and, were calculated using the value of $\sigma_{\mathrm{in}}$, $\sigma_{\mathrm{eq}}$ and the strain (i.e., deformation due to applied stress) of the cartilage. The cartilage section was immersed PBS at all times during the data acquisition at the room temperate (~22 °C).

### 4.3. Statistical Analyses

Multiple-group statistical comparisons between ICRS grades-I, II and III were assessed by nonparametric Kruskal-Wallis ANOVA test in Matlab^®^ (The MathWorks, 2014, Natick, MA, USA). The Shapiro-Wilk’s test showed non-normal distribution of the data and therefore, nonparametric test was performed. In nonparametric multiple-group pairwise comparisons, the value of *p* < 0.05 (*), *p* < 0.01 (**) and *p* < 0.001 (***) were considered indicative of statistical significance at the given significance level. Box plots display median values and interquartile ranges.

### 4.4. Histological Assessment

After data acquisition of the stress relaxation with each ICRS grade cartilage section, tissue section was stored in 10% neutral-buffered formalin (NBF), and further processed for histological evaluation. Histological assessment was performed using the Osteoarthritis Research Society International (OARSI) grading system [35]. Based on morphology, H&E and Safranin-O color evaluation, each tissue sample was assigned an OARSI grade.

## 5. Conclusions

This work focused on characterization of the different stages (i.e., ICRS grades-I, II, and III) of human osteoarthritic cartilage. Applying polymer dynamics theory, a reduction in stress-relaxation time and stretch-exponent parameter were observed in advanced OA (i.e., ICRS grade-III). In addition, a loss in compressive Young’s modulus and reduced mechanical vitality were also observed in ICRS grade-III. The reduction in PG content could be the reason for the loss in compressive Young’s modulus and faster stress relaxation in the ICRS grade-III osteoarthritic cartilage. The stretch-exponent parameter indicated a significant loss of hyaluronic acid accompanied by the loss of PG in ICRS grade-III. Furthermore, in numerical modeling of osteoarthritic articular cartilage, the experimental data of this work can be utilized as input parameters to minimize the error between model and experiments. The future work aims to reveal how the change in mechanical behavior plays a role in pathogenesis of early stage OA. More detailed information about cartilage biomechanics are essential and have practical importance for not only understanding cartilage mechanics but also for enhancing the knowledge of disease progression.

## Figures and Tables

**Table 1:** The characteristic values of osteoarthritic cartilage ICRS grades—I, II and III.

Sample	Stress-Relaxation Time Constant (*τ*)	Instantaneous Young’s Modulus i.e., Y_in_ (at *t* = 0 s)	Equilibrium Young’s Modulus i.e., Y_eq_ (at *t* = 1180 s)	Ratio (Y_in_/Y_eq_)	Stretching Exponent (*β*)	Histological Grade (OARSI)
ICRS grade-I						
#Section-1	211.2	6.816	6.816	2.236544	0.6776	1
#Section-4	136	3.426691	3.426691	3.228219	0.6013	2
#Section-9	382.4	4.559351	4.559351	1.684617	0.9331	1
#Section-11	157.6	3.7488	3.7488	2.005482	0.5947	2
#Section-13	187.6	5.797113	5.797113	1.996035	0.631	1
#Section-16	185.9	3.360478	3.360478	2.039817	0.6165	2
#Section-19	110	4.172465	4.172465	2.493802	0.5537	1
#Section-21	132.5	3.910431	3.910431	2.713876	0.5578	1
ICRS grade-II						
#Section-3	116.4	3.997535	3.997535	2.920333	0.6004	2
#Section-5	172.3	5.50605	5.50605	2.475084	0.6501	2
#Section-7	151.3	4.317236	4.317236	2.681396	0.6163	1
#Section-12	64.82	4.065479	4.065479	2.297605	0.4867	2
#Section-14	172	3.928383	3.928383	2.129691	0.5743	2
#Section-17	106.1	3.736842	3.736842	2.126649	0.5062	2
#Section-20	145.3	3.421194	3.421194	2.116525	0.6046	2
#Section-24	161.3	3.685106	3.685106	1.842395	0.6004	1
ICRS grade-III						
#Section-2	64.28	1.497124	0.443423	3.376287	0.4393	3
#Section-6	43.52	2.203448	0.712712	3.091636	0.4363	3
#Section-8	55.04	4.301901	1.327796	3.239881	0.4687	4
#Section-10	56.87	1.687635	0.586799	2.876001	0.4422	3
#Section-15	43.9	2.677714	0.997392	2.684714	0.4584	3
#Section-18	111.6	2.870444	1.288061	2.228499	0.5566	3
#Section-22	72.66	3.397942	1.231759	2.758608	0.4996	4
#Section-23	27.74	1.927954	0.496422	3.883696	0.4236	3

**Table 2:** ICRS classification of osteoarthritic cartilage sample. L and M represent lateral and medial femoral condyle section of articular cartilage respectively.

Cartilage Section (Total *n* = 24)	Assignment of ICRS Grdae		
	Grade-I (*n* = 8)	Grade-II (*n* = 8)	Grade-III (*n* = 8)
Patient 1	Section-1 (L)	x	x
Patient 2	x	x	Section-2 (M)
Patient 3	x	Section-3 (L)	x
Patient 4	Section-4 (M)	Section-5 (M)	Section-6 (L)
Patient 5	x	Section-7 (M)	Section-8 (L)
Patient 6	Section-9 (L)	x	Section-10 (M)
Patient 7	Section-11 (L)	Section-12 (M)	x
Patient 8	Section-13 (L)	Section-14 (L)	Section-15 (M)
Patient 9	Section-16 (M)	x	x
Patient 10	x	Section-17 (M)	Section-18 (M)
Patient 11	Section-19 (L)	Section-20 (M)	x
Patient 12	Section-21 (L)	x	Section-22 (M)
Patient 13	x	x	Section-23 (M)
Patient 14	x	Section-24 (L)	x
